# Efficacy and safety of radiofrequency ablation versus cryoballoon ablation for persistent atrial fibrillation: a systematic review and meta-analysis of randomized controlled trials

**DOI:** 10.1186/s43044-024-00518-x

**Published:** 2024-07-08

**Authors:** Ahmed Mazen Amin, Ahmad Nawlo, Ahmed A. Ibrahim, Ahmed Hassan, Alhassan Saber, Mohamed Abuelazm, Basel Abdelazeem

**Affiliations:** 1 Faculty of Medicine, Mansoura University, Mansoura, Egypt; 2 Division of Infectious Diseases, Brigham and Women’s Hospital- Harvard Medical School, Boston, MA 02115 USA; 3 Faculty of Medicine, Menoufia University, Saad Zaghloul St., Shibin El-Kom, 32511 Menoufia Governorate Egypt; 4 Faculty of Medicine, October 6 University, Giza, Egypt; 5 Faculty of Medicine, Minia University, Minya, Egypt; 6 Faculty of Medicine, Tanta University, Tanta, Egypt; 7 Department of Cardiology, West Virginia University, Morgantown, WV USA; 8 Department of Cardiology, Suez Medical Complex, Ministry of Health and Population, Suez, Egypt

**Keywords:** Ablation, Atrial fibrillation, Pulmonary vein isolation, Arrhythmia, Review

## Abstract

**Background:**

Persistent Atrial Fibrillation (PeAF) is a challenging case for rhythm control modalities. Catheter ablation is the mainstay in PeAF management; however, data regarding the comparative safety and efficacy of cryoballoon ablation (CBA) versus radiofrequency ablation (RFA) for PeAF is still limited. We aim to compare the safety and efficacy of CBA versus RFA for PeAF ablation.

**Methods:**

We conducted a systematic review and meta-analysis synthesizing randomized controlled trials (RCTs), which were retrieved by systematically searching PubMed, EMBASE, Web of Science, SCOPUS, and Cochrane through October 2023. RevMan version 5.4 software was used to pool dichotomous data using risk ratio (RR) and continuous data using mean difference (MD) with a 95% confidence interval (CI). PROSPERO ID: CRD42023480314.

**Results:**

Three RCTs with 400 patients were included. There was no significant difference between RFA and CBA regarding AF recurrence (RR: 0.77, 95% CI [0.50, 1.20], *P* = 0.25), atrial tachycardia or atrial flutter recurrence (RR: 0.54, 95% CI [0.11, 2.76], *P* = 0.46), and any arrhythmia recurrence (RR: 0.96, 95% CI [0.70, 1.31], *P* = 0.80). CBA was significantly associated with decreased total procedure duration (MD: − 45.34, 95% CI [− 62.68, − 28.00], *P* < 0.00001), with no significant difference in fluoroscopy duration (MD: 3.59, 95% CI [− 5.13, 12.31], *P* = 0.42). Safety parameters were similar in both groups, including the incidence of any complications, phrenic nerve palsy (RR: 2.91 with 95% CI [0.31, 27.54], *P* = 0.35), access site complications (RR: 0.33 with 95% CI [0.05, 2.03], *P* = 0.23), and pericardial effusion.

**Conclusions:**

In PeAF catheter ablation, CBA is comparable to RFA in terms of safety and efficacy. Also, CBA is associated with a shorter total procedure duration.

**Supplementary Information:**

The online version contains supplementary material available at 10.1186/s43044-024-00518-x.

## Background

In the last 50 years, the prevalence and incidence of atrial fibrillation (AF) have been on the rise, attributed to increasing global life expectancy [1]. Alongside this trend, diagnostic and treatment approaches for AF have been evolving. Despite this progress, the exact etiology of AF remains incompletely understood, though substantial evidence points to ectopic electrical activity in and around the pulmonary veins (PV). As a result, PV isolation has become the cornerstone of catheter-based AF treatment [2–4].

Currently, two primary approaches are employed to achieve PV isolation. The first is radiofrequency ablation (RFA), which utilizes radio waves to create a conduction block, enhancing the isolation of the PV. The second approach is cryoballoon ablation (CBA), where liquified nitrogen is used to scar arrhythmic tissue [5]. Numerous studies have compared the efficacy of RFA and CBA in treating paroxysmal AF, with many demonstrating no significant clinical outcome differences. Furthermore, several studies reported no difference in the recurrence rate of atrial tachyarrhythmia [6–8]. However, for patients with persistent atrial fibrillation (PeAF), which is considered very challenging to treat and has a high recurrence rate following ablation, limited data are available regarding the effectiveness and recurrence rate of RFA vs. CBA in this population [9, 10]. The most recent randomized controlled trial (RCT), conducted by Mililis et al., suggested that RFA is comparable to CBA regarding recurrence rate. Still, it highlighted that CBA is associated with decreased procedural duration in patients with PeAF [11].

A systematic review exploring the efficacy and safety of CBA vs. RFA was published in 2022, including one RCT and nine observational studies [12]. However, two new RCTs comparing RFA vs. CBA have been recently published, which we included in this paper to be systematically reviewed [11, 13]. In this systematic review, we aim to evaluate the efficacy and safety of RFA vs. CBA regarding arrhythmia recurrence, freedom from arrhythmia, total procedure time, ablation time, fluoroscopy time, and the need for repeated ablations.

## Methods

### Protocol registration

The current study was rigorously constructed to comply with the Preferred Reporting Items for Systematic Reviews and Meta-Analyses: The PRISMA Statement guidelines for systematic reviews and meta-analysis [14] and the Cochrane Handbook for Systematic Reviews and Meta-Analysis guidelines [15]. This meta-analysis was prospectively registered in the International Prospective Register of Systematic Reviews (PROSPERO) under ID: CRD42023480314.

### Data sources & search strategy

A comprehensive literature search was conducted through PubMed, Web of Science, Cochrane Library, Scopus, and Embase up to October 2023. A systematic approach was employed to include only RCTs. The search methodology executed involved the application of the following search terms: (“Radiofrequency Ablation”, “Radio Frequency Ablation”, “Radiofrequency”, “Radio Frequency”) AND (“Cryoballoon ablation”, “Cryoballoon”) AND (“persistent atrial fibrillation”, “persistent afib”, “persistent AF”), further details are highlighted in (Additional file 1: Table S1).

### Eligibility criteria

We included RCTs following Population, Intervention, Comparison, and Outcomes (PICO) criteria: population (P): patients with PeAF undergoing first ablation; intervention (I): RFA; control (C): CBA; outcome (O): our primary outcomes were AF recurrence, recurrence of atrial tachycardia/atrial flutter, and arrhythmia recurrence, while secondary outcomes were: total procedure time, fluoroscopy time, repeated ablation, and safety outcomes (any complications, phrenic nerve palsy, access site complications, and pericardial tamponade), while exclusion criteria involved observational studies, abstracts, and animal studies.

### Study selection

After saving the records from searching the previously mentioned databases using our search strategy in Covidence, two reviewers (A.H. and A.S.) independently screened the title and abstract, then the full text of the resulting records per our previously mentioned eligibility criteria. Any discrepancies were solved by a third author (A.M.A. and M.A.).

### Data extraction

Two reviewers independently extracted the data of the included studies in a Microsoft Excel sheet (A.H. and A.S.). Any discrepancies were solved by a third author (A.M.A. and M.A.). Extracted data encompassed study characteristics (study design, country, number of centers, the total number of patients in the study, details about both the radiofrequency and the cryoballoon ablation groups, main inclusion criteria, and the span of follow-up); baseline patient characteristics (number of patients in the radiofrequency and cryoballoon ablation groups, gender (male), age (years), body mass index (BMI), CHA_2_DS_2_VASc score, left ventricular ejection fraction (LVEF), left atrium diameter, medications history (beta-blockers and amiodarone), and comorbidities (hypertension, diabetes mellitus, coronary artery disease, obstructive sleep apnea, dyslipidemia); and outcomes (AF recurrence, recurrence of atrial tachycardia/atrial flutter, arrhythmia recurrence, repeated ablation, total procedure time, and fluoroscopy time, and safety outcomes (any complications, phrenic nerve palsy, access site complications, and pericardial tamponade)). Any discrepancies were resolved through discussion.

### Risk of bias and certainty of evidence

We utilized the revised Cochrane risk-of-bias tool for randomized trials (RoB 2) [16] to evaluate the risk of bias in the included RCTs. This evaluation encompassed an assessment of the randomization process, concealment of the allocation sequence, deviations from the intended interventions, utilization of appropriate analysis to estimate the effect of assignment to intervention, measurement of the outcome, selection of the reported results, and overall risk of bias. The assessment of the methodological quality of the studies was classified as either low risk, with some concerns, or high risk of bias.

M.A. used the Grading of Recommendations Assessment, Development, and Evaluation (GRADE) guidelines [17, 18] to assess the certainty of evidence for each outcome.

### Statistical analysis

We used RevMan v5.3 to conduct the statistical analysis [19]. For pooling the results of dichotomous outcomes, we used the risk ratio (RR), while for the continuous outcomes, we used the mean difference (MD), both with a 95% confidence interval (CI). We performed both the Chi-square and I-square tests to evaluate heterogeneity, where the Chi-square test detects the presence of heterogeneity, and the I-square test evaluates its degree. I-square was interpreted in accordance with the Cochrane Handbook (chapter 9) [15] as follows: heterogeneity is not significant for 0–40 percent, moderate for 30–60 percent, substantial for 50–90 percent, and considerable for 75–100 percent. We considered an alpha level below 0.1 for the Chi-square test to detect the significant heterogeneity. Leave-one-out sensitivity analysis was employed to resolve the heterogeneity by removing each trial one time from the pooled analyzed studies.

## Results

### Search results and study selection

**Fig. 1:**
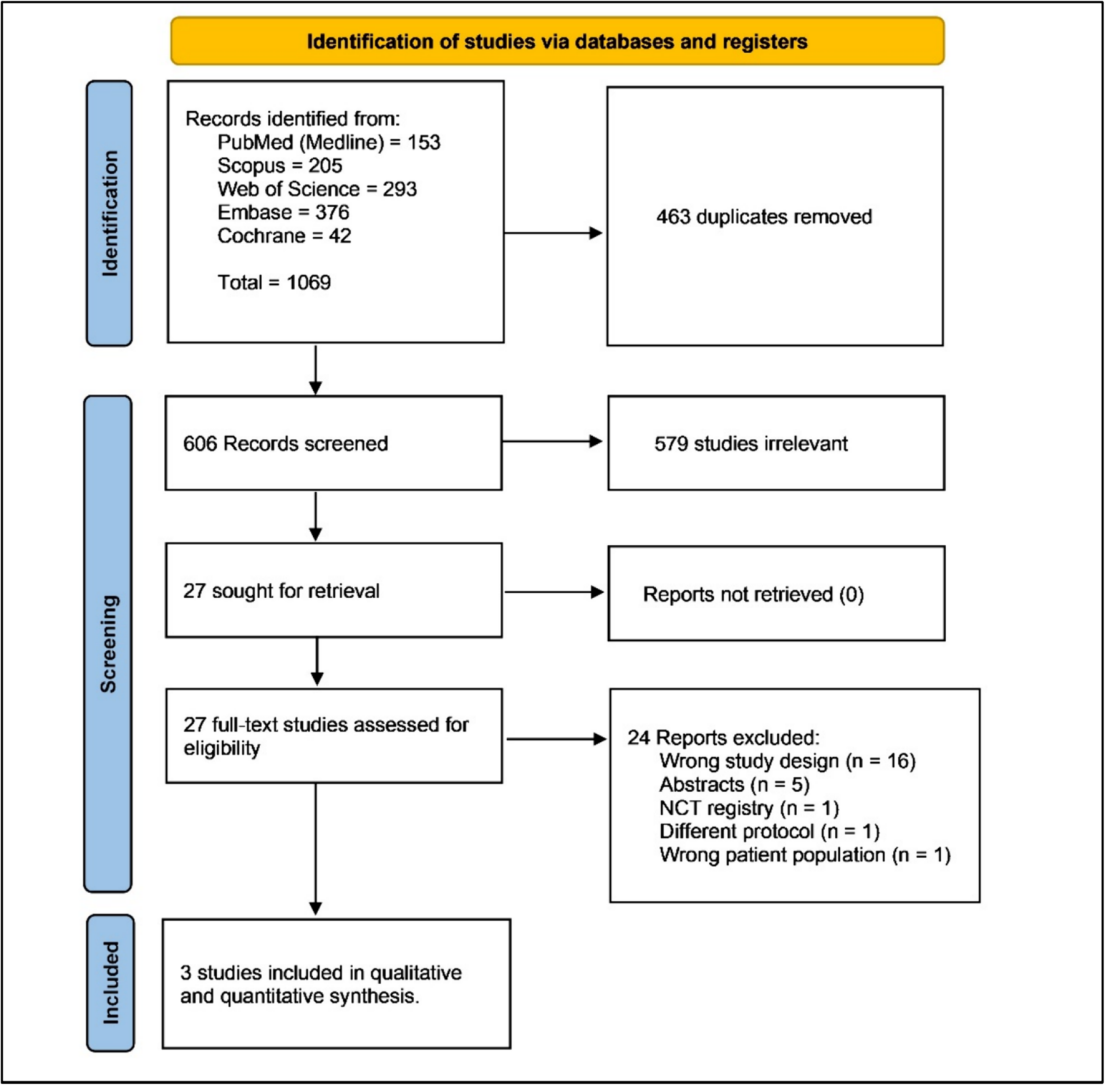
PRISMA flow chart of the screening process

In the pursuit of research, 1,069 articles emerged (153 in PubMed, 293 in Web of Science, 376 in Embase, 42 in Cochrane, and 205 in Scopus). After excluding 463 duplicates, we scrutinized the remaining 606. Following a title and abstract screening, 579 articles were dismissed. Upon full-text review, 24 more were eliminated. Consequently, three RCTs were included in both qualitative and quantitative synthesis. The literature search flow diagram is depicted in (Fig. 1). Of note, we excluded one RCT [20] from our analysis due to the difference in the protocol used in the cryotherapy arm, as they used a tandem focal cryoablation catheter in addition to the cryoballoon, which was significantly different from the other included RCTs.

### Characteristics of included studies

We included three RCTs [11, 13, 21] with 400 patients; 232 patients were in the RFA arm, and 168 patients were in the CBA. More details about the trials and participants characteristics are summarized in (Tables 1, 2, Additional file 1: Tables S1, and S2).

**Table 1:** Summary characteristics of the included RCTs

Study ID	Study design	Country	Total participants	Main inclusion criteria	Primary outcome	Follow-up duration
Baimbetov et al. [13]	Randomized controlled single-center trial	Kazakhstan	100	Patients with persistent AF in the last six months before inclusion in the study, for whom at least two antiarrhythmic drugs of class I to III were not effective	AF, atrial flutter, atrial tachycardia, and PLUS freedom from chronic treatment failure, which is determined by the absence of any detectable arrhythmia after the blanking period	36 months
Mililis et al. [11]	Randomized controlled single-center trial	Greece	199	Age > 18 years, symptomatic persistent AF, denovo CBA for AF and willingness to participate in the study	Arrhythmia relapse in the first three months and 3 months to 12 months postprocedural period	12 months
Shi et al. (NO-PERSAF)[21]	Randomized controlled multi-center trial	Norway	101	patients who underwent PV isolation as the first ablation procedure for symptomatic persistent AF (lasting for > seven days, but < 12 months) or long-standing persistent AF (lasting for > 12 months) refractory to at least one antiarrhythmic drug	Any documented recurrent atrial tachyarrhythmia (ATA) lasting longer than 30 s following a 3-month blanking period	12 months

*AF* Atrial fibrillation

**Table 2:** Baseline characteristics of the participants

Study ID	Number of patients in each group		Age (Years), Mean (SD)		Gender (male) N.%		BMI, Mean (SD)		CHA_2_DS_2_VASc score, Mean (SD)		Echocardiographic characteristics N. (%)			
	Radiofrequency ablation	Cryoballoon ablation	Radiofrequency ablation	Cryoballoon ablation	Radiofrequency ablation	Cryoballoon ablation	Radiofrequency ablation	Cryoballoon ablation	Radiofrequency ablation	Cryoballoon ablation	LVEF, mean (SD)		LA diameter (mm), mean (SD)	
											Radiofrequency ablation	Cryoballoon ablation	Radiofrequency ablation	Cryoballoon ablation
Baimbetov et al. [13]	50	50	61.6 (6.5)	61.3 (10.2)	29 (58)	31 (62)	NA	NA	0.9 (0.6)	1 (0.5)	54 (6)	59 (5)	39 (7)	41 (5)
Mililis et al. [11]	133	66	60.22 (9.87)	62.74 (9.09)	111 (83.3)	51 (77.3)	30.07 (5.28)	33.07 (3.11)	1.61 (1.31)	1.71 (1.37)	55.15 (7.10)	55.70 (6.16)	44.89 (4.79)	43.55 (4.74)
Shi et al. (NO-PERSAF)[21]	49	52	64.0 (8.7)	62.4 (8.4)	35 (71.4)	45 (86.5)	28.8 (4.5)	29.6 (4.7)	1.46 (0.73)	1.25 (0.86)	56.8 (8.1)	56.0 (7.2)	44 (7)	46 (6)

*SD* Standard deviation, *BMI* Body mass index, *LVEF* Left ventricular ejection fraction, *LA* Left atrium, *NA* Not available

### Risk of bias and certainty of evidence

**Fig. 2:**
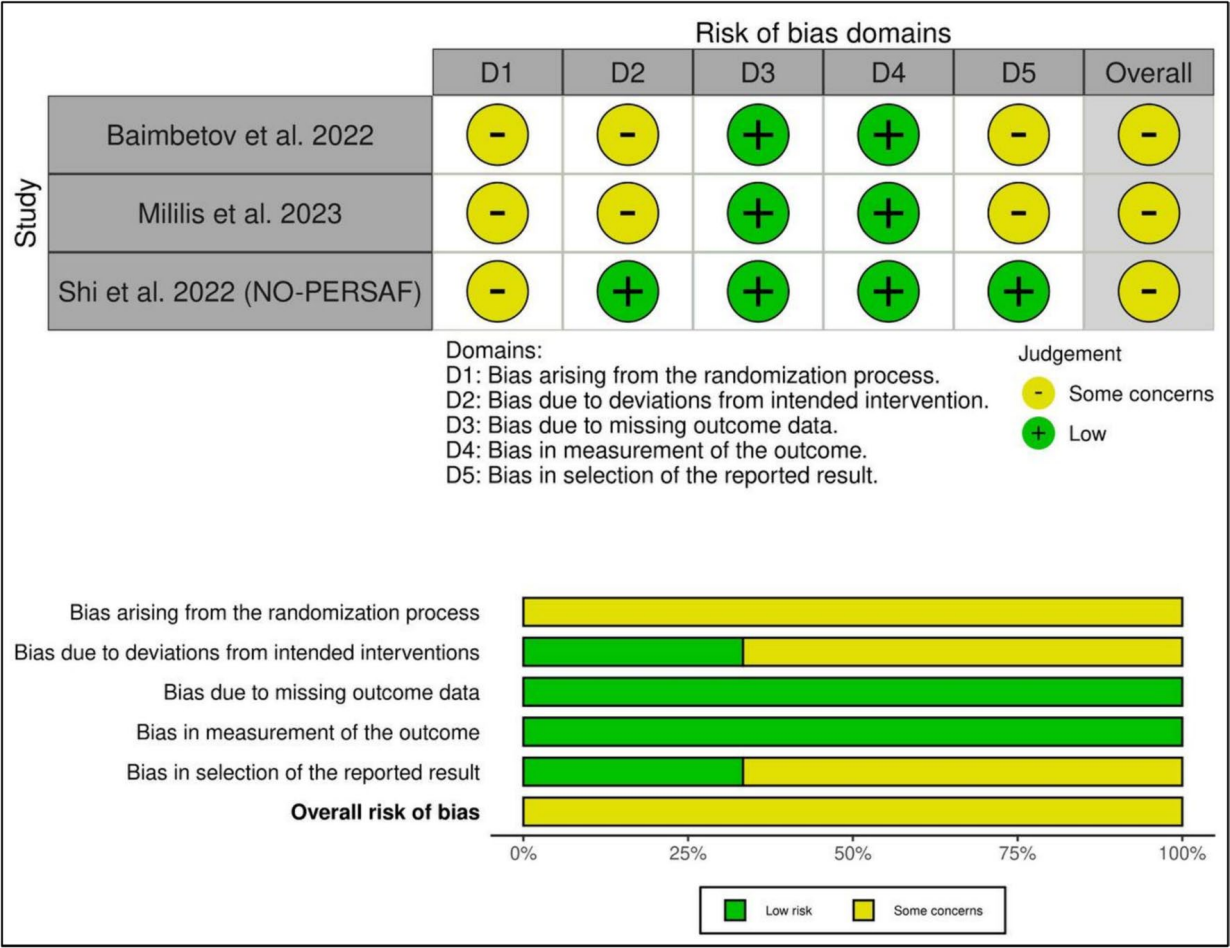
Quality assessment of risk of bias in the included trials. The upper panel presents a schematic representation of risks (low = green, unclear = yellow, and high = red) for specific types of biases of each study in the review. The lower panel presents risks (low = green, unclear = yellow, and high = red) for the subtypes of biases of the combination of studies included in this review

Upon assessing the risk of bias by the ROB 2 tool, all the RCTs had some concerns overall. The RoB results are shown in **(**Fig. 2**),** and more details about the author's judgments of the risk of bias assessment are outlined in (Additional file 1: Table S4). The certainty of evidence is outlined in a GRADE evidence profile (Table 3).

**Table 3:** GRADE evidence profile

Certainty assessment							Summary of findings				
Participants (studies) Follow-up	Risk of bias	Inconsistency	Indirectness	Imprecision	Publication bias	Overall certainty of evidence	Study event rates (%)		Relative effect (95% CI)	Anticipated absolute effects	
							With [Radiofrequency Ablation]	With [Cryoballoon Ablation]		Risk with [Radiofrequency Ablation]	Risk difference with [Cryoballoon Ablation]
*AF recurrence*											
300 (2 RCTs)	Serious^a^	Not serious	not Serious	Extremely serious^b^	None	⨁◯◯◯ Very low	42/182 (23.1%)	24/118 (20.3%)	RR 0.77 (0.50 to 1.20)	231 per 1,000	53 fewer per 1,000 (from 115 fewer to 46 more)
*Recuurence of atrial tachycardia/atrial flutter,*											
201 (2 RCTs)	Serious^a^	Very serious^c^	Not serious	Extremely serious^b^	None	⨁◯◯◯ Very low	23/99 (23.2%)	17/102 (16.7%)	RR 0.54 (0.11 to 2.76)	232 per 1,000	107 fewer per 1,000 (from 207 fewer to 409 more)
*Arrhythmia recurrence*											
400 (3 RCTs)	Serious^a^	Not serious	Not serious	Extremely serious^b^	None	⨁◯◯◯ Very low	68/232 (29.3%)	49/168 (29.2%)	RR 0.96 (0.70 to 1.31)	293 per 1,000	12 fewer per 1,000 (from 88 fewer to 91 more)
*Repeated ablation*											
299 (2 RCTs)	Serious^a^	Not serious	Not serious	EXTREMELY serious^b^	None	⨁◯◯◯ Very low	34/183 (18.6%)	25/116 (21.6%)	RR 1.19 (0.74 to 1.92)	186 per 1,000	35 more per 1,000 (from 48 fewer to 171 more)
*Total procedure time (min)*											
400 (3 RCTs)	Serious^a^	Very serious^c^	Not serious	Not serious	None	⨁◯◯◯ Very low	232	168	-	The mean total procedure time (min) was 0	MD 45.34 lower (62.68 lower to 28 lower)
*Fluoroscopy time (min)*											
400 (3 RCTs)	Serious^a^	Very serious^c^	Not serious	Not serious	None	⨁◯◯◯ Very low	232	168	-	The mean fluoroscopy time (min) was 0	MD 3.59 higher (5.13 lower to 12.31 higher)
*Safety—Any complications*											
300 (2 RCTs)	Serious^a^	Not serious	Not serious	Extremely serious^b^	None	⨁◯◯◯ Very low	4/182 (2.2%)	1/118 (0.8%)	RR 0.40 (0.07 to 2.42)	22 per 1,000	13 fewer per 1,000 (from 20 fewer to 31 more)
*Safety—Phrenic nerve palsy*											
400 (3 RCTs)	Serious^a^	Not serious	Not serious	Extremely serious^b^	None	⨁◯◯◯ Very low	0/232 (0.0%)	2/168 (1.2%)	RR 2.91 (0.31 to 27.54)	0 per 1,000	0 fewer per 1,000 (from 0 to 0 fewer)
*Safety—Access site complication*											
400 (3 RCTs)	Serious^a^	Not serious	Not serious	Extremely serious^b^	None	⨁◯◯◯ Very low	4/232 (1.7%)	1/168 (0.6%)	RR 0.33 (0.05 to 2.03)	17 per 1,000	12 fewer per 1,000 (from 16 fewer to 18 more)
*Safety—Pericardial effusion*											
300 (2 RCTs)	Serious^a^	Not serious	Not serious	Extremely serious^b^	None	⨁◯◯◯ Very low	2/182 (1.1%)	0/118 (0.0%)	RR 0.45 (0.05 to 4.14)	11 per 1,000	6 fewer per 1,000 (from 10 fewer to 35 more)

*CI* Confidence interval, *MD* Mean difference, *RR* risk ratio

^a^All trials had overall some concerns of bias

^b^Wide confidence interval that does not exclude the appreciable harm/benefit + low number of events

^c^Considerable heterogeneity (I^2^ > 75%)

### Primary outcomes

**Fig. 3:**
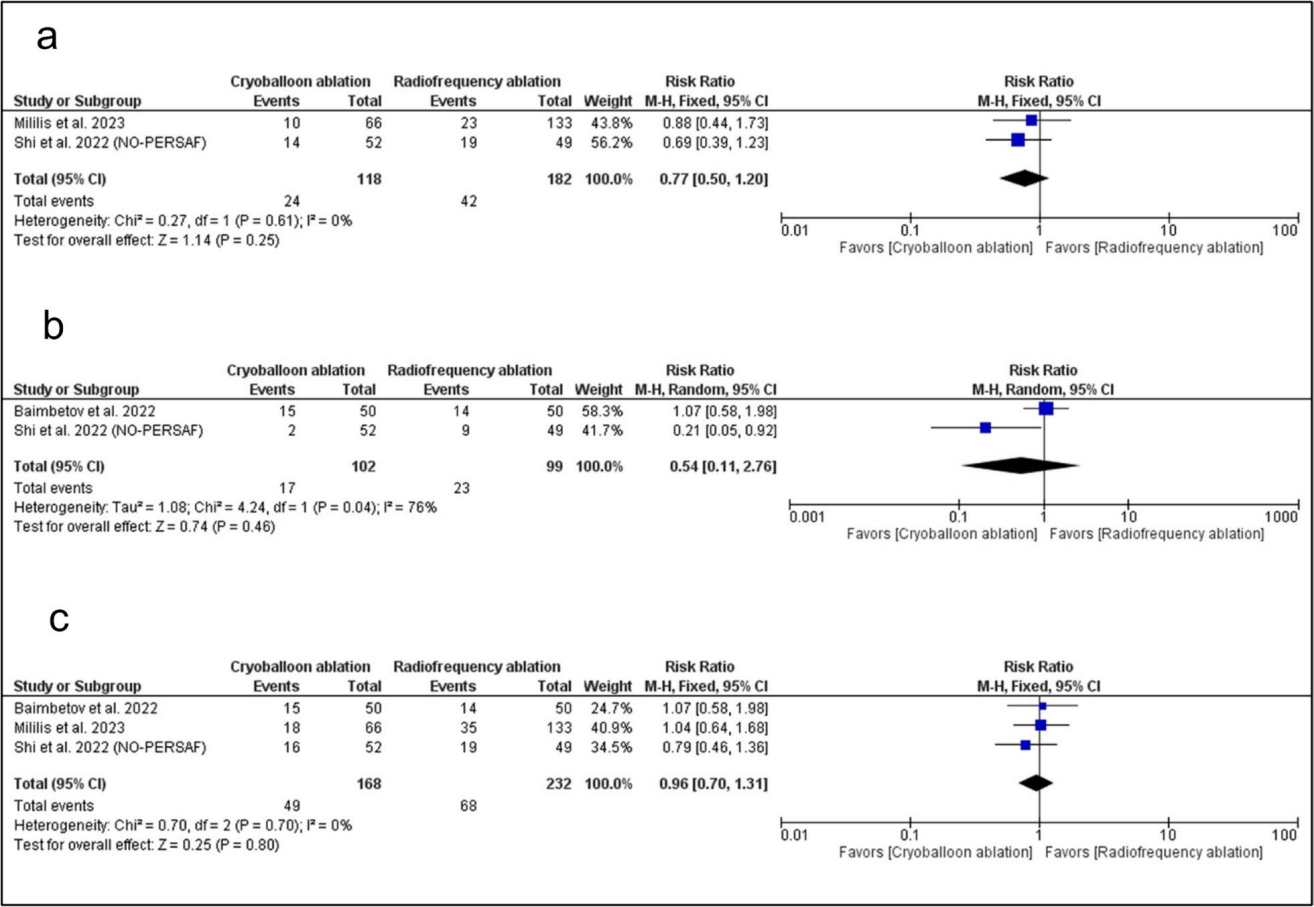
Forest plot of the primary outcomes **A** atrial fibrillation recurrence, **B** atrial tachycardia/atrial flutter recurrence, **C** Any arrhythmia recurrence. RR: risk ratio, CI: confidence interval

There was no significant difference between RFA ablation and CBA ablation in the incidence of AF recurrence (RR: 0.77 with 95% CI [0.50, 1.20], *P* = 0.25) (Fig. 3A), atrial tachycardia or atrial flutter recurrence (RR: 0.54 with 95% CI [0.11, 2.76], *P* = 0.46) (Fig. 3B), and arrhythmia recurrence (RR: 0.96 with 95% CI [0.70, 1.31], *P* = 0.80) (Fig. 3C). The pooled studies were homogenous in AF recurrence (I^2^ = 0%, *P* = 0.61) and the incidence of arrhythmia recurrence (I^2^ = 0%, *P* = 0.70). However, pooled studies were heterogeneous in the incidence of atrial tachycardia or atrial flutter recurrence (I^2^ = 76%, *P* = 0.04). Sensitivity analysis was not applicable regarding the incidence of atrial tachycardia or atrial flutter.

### Secondary outcomes

#### Procedural outcomes

**Fig. 4:**
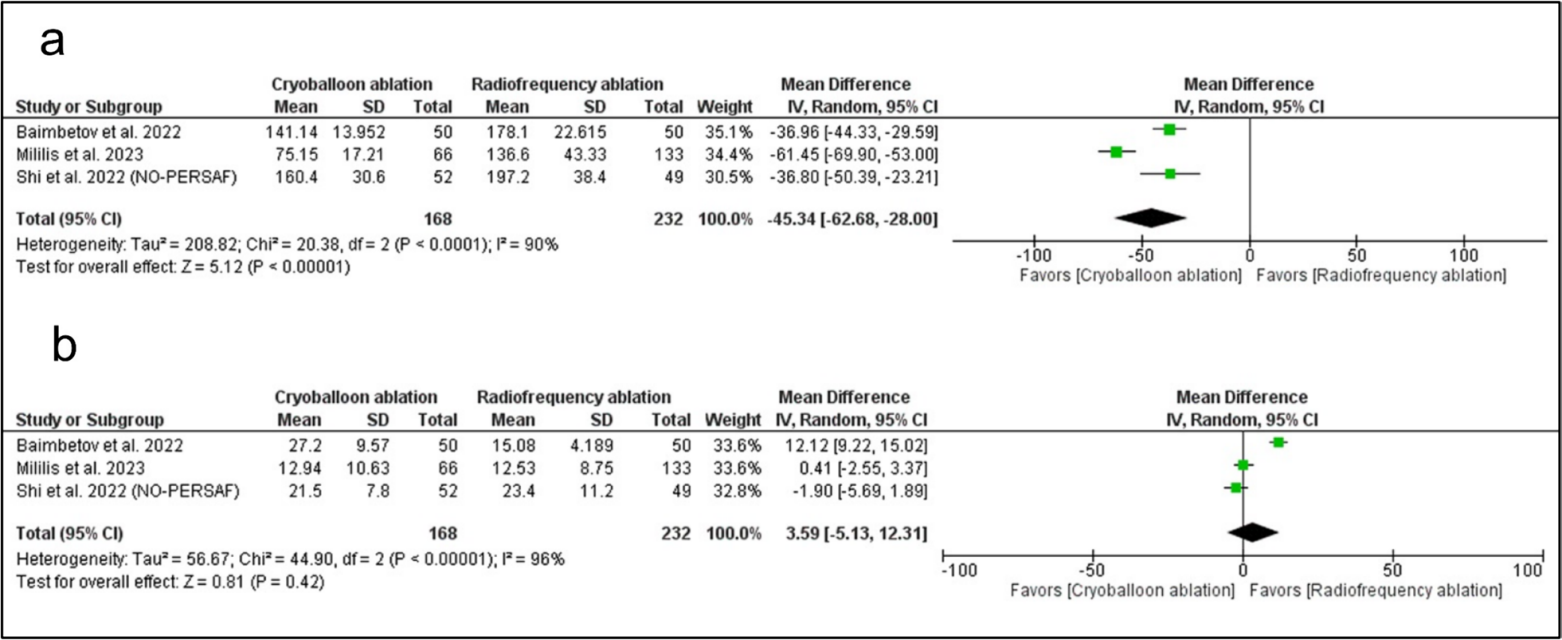
Forest plots of the secondary procedural outcomes **A** total procedure time, **B** fluoroscopy time, RR: risk ratio, CI: confidence interval

CBA was significantly associated with decreased total procedure time (MD: − 45.34 with 95% CI [− 62.68, − 28.00], *P* < 0.00001) (Fig. 4A). However, there was no significant difference between RFA and CBA in fluoroscopy time (MD: 3.59 with 95% CI [− 5.13, 12.31], *P* = − 0.42) (Fig. 4B). Additionally, there was no significant difference between RFA and CBA in the incidence of repeated ablation (RR: 1.19 with 95% CI [0.74, 1.92], *P* = 0.47) (Additional file 1: Fig. S1).

The pooled studies were homogenous in the incidence of repeated ablation (I^2^ = 0%, *P* = 0.44). However, the pooled studies were heterogeneous in total procedure time (I^2^ = 90%, *P* < 0.0001) and fluoroscopy time (I^2^ = 96%, *P* < 0.00001). Regarding total procedure time, heterogeneity was best resolved by excluding Mililis et al. 2023 (I^2^ = 0%, *P* = 0.98). Regarding fluoroscopy time, heterogeneity was best resolved by excluding Baimbetov et al. 2022 (I^2^ = 0%, *P* = 0.35). (Additional file 1: Table S5).

#### Safety outcomes

**Fig. 5:**
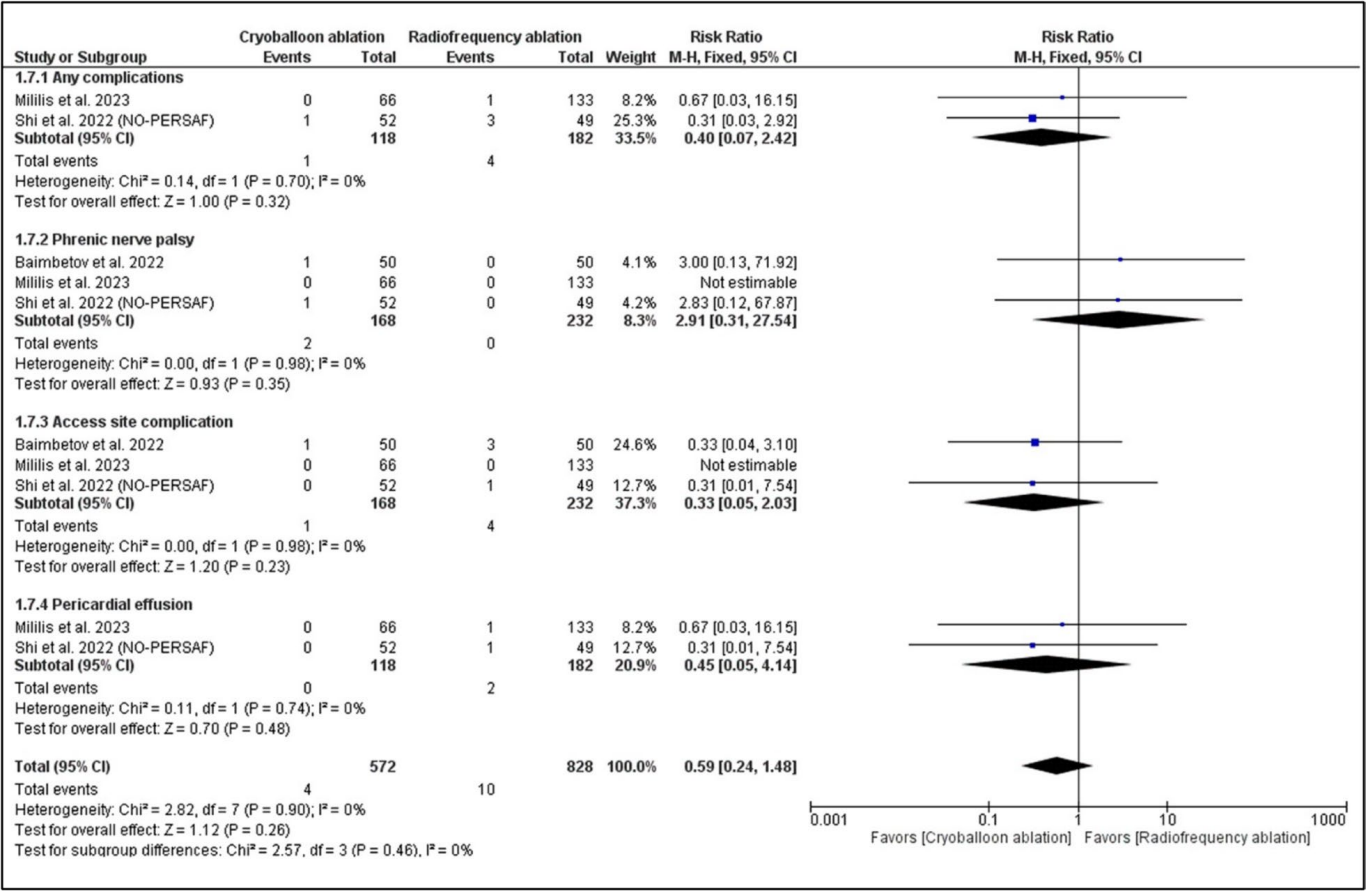
Forest plots of the safety outcomes, RR: risk ratio, CI: confidence interval

There was no significant difference between RFA and CBA in the incidence of any complications (RR: 0.40 with 95% CI [0.07, 2.42], *P* = 0.32), the incidence of phrenic nerve palsy (RR: 2.91 with 95% CI [0.31, 27.54], *P* = 0.35), the incidence of access site complications (RR: 0.33 with 95% CI [0.05, 2.03], *P* = 0.23), and the incidence of pericardial effusion (RR: 0.45 with 95% CI [0.05, 4.14], *P* = 0.48) (Fig. 5).

The pooled studies were homogenous in the incidence of any complications (I^2^ = 0%, *P* = 0.70), phrenic nerve palsy (I^2^ = 0%, *P* = 0.98), access site complications (I^2^ = 0%, *P* = 0.98), and pericardial effusion (I^2^ = 0%, *P* = 0.74).

## Discussion

In this systematic review, we examined the safety and efficacy of CBA and RFA for PeAF. Our study revealed that CBA was comparable to RFA regarding AF and arrhythmia recurrence. However, CBA was linked to a notably shorter procedural time with no significant difference in fluoroscopy time and complication rate compared to RFA.

Second-generation CBA has recently emerged as a new technique in interventional cardiology, addressing limitations observed in the first generation. It achieves high cooling efficiency and more extensive lesion formation, resulting in a more homogeneous freezing effect [22]. A recent study has proved the efficacy of CBA treating PeAF [23, 24]. While the FIRE AND ICE study and the CIRCA-DOSE study have explored the efficacy and safety of CBA and RFA in managing paroxysmal AF, both showed no superiority of RFA over CBA in the clinical outcome for patients with the paroxysmal pattern [25, 26], there remains a scarcity of studies comparing both techniques specifically in PeAF cohort.

Catheter ablation offers numerous advantages over antiarrhythmic drugs, particularly in the treatment of PeAF, a condition known for its high refractory rate [27]. The NO-PERSAF study [21], an RCT conducted in Norway, aimed to compare the efficacy of CBA and RFA in treating PeAF. The study results revealed no significant difference between CBA and RFA in terms of the recurrence rate of arrhythmia. This finding aligns with the conclusions drawn by Millis et al. and Baimbetov et al. [11, 13]. These findings’ similarities may be because the same procedural techniques have been used in three studies and the protocol's resemblances, even though Baimbetov et al. [13] considered a more extensive follow-up period. Our meta-analysis, incorporating data from these three studies, further reinforces this outcome, indicating no statistically significant difference in the recurrence rates of arrhythmia, atrial flutter, or AF.

In our study, we explored the procedure duration for both interventions along with the fluoroscopy time. Our analysis uncovered a noteworthy finding that the CBA arm was found to have significantly less procedure time with a mean difference of − 45 min [− 62.68, − 28.00]. This goes in line with all the included RCTs [11, 13, 21]. However, it is essential to acknowledge that Shi et al. used three-dimensional mapping, and Baimbetov et al. had a 30-min evaluation period that may lead to a longer time in both groups [21]. Nevertheless, as experience accumulates, the overall ablation time decreases. On the other hand, Millis et al. and Shi et al. studies did not observe a significant difference in the fluoroscopy time [11, 21]. In contrast to Bamibetov et al. [13], whose study demonstrated that RFA was superior to CBA, showcasing less fluoroscopy time; this may be attributed to the fact that the ablation process was performed following the three-dimensional reconstruction of the LA, which led to less use of X-ray control. However, our paper did not conclusively affirm this finding, as no statistical significance was observed in the fluoroscopy time.

Similar to any medical intervention, there are risks of adverse events. In contrast, in the FREEZE AF study [6], major complication rates until discharge were notably low in both CBA and RFA groups. However, the study observed that the primary significant complication in the CBA group was phrenic nerve palsy, persisting until discharge (1.1%). In contrast, the RFA group exhibited a notable incidence of access site complications (4.3%), which could be attributed to a potential selection bias given the comparatively higher illness severity of patients in this group. Nevertheless, these numerical differences were clinically insignificant. The RCTs included in our analysis revealed no discernible differences in outcomes such as phrenic nerve palsy, access site complications, and overall complications [11, 13, 21]. This disparity may be attributed to the utilization of second-generation CB technology and the application of contact force sensing catheters in the study cohort, while in the FREEZE AF study, the CBA group was treated with first-generation CB, recognized for its lower efficacy compared to the second-generation. Our paper further corroborates these findings by demonstrating no significant distinctions between the two groups regarding any complications, phrenic nerve palsy, access site complications, and pericardial effusion.

### Strengths

In this systematic review, we investigated the efficacy and safety of CBA and RFA in patients with PeAF. All the included studies were RCTs, and no observational or reviews were included. This type of selection enhances the credibility of our study by relying on rigorous experimental designs that are considered robust in scientific research.

### Limitations

This paper has several limitations. The inclusion and exclusion criteria were met by only three randomized controlled trials, potentially impacting the robustness of the findings due to the limited sample size and an increased risk of bias. Furthermore, the constrained number of included studies and the high heterogeneity of various variables prevented the feasibility of conducting a subgroup analysis. The high heterogeneity could be justified by the differences in inclusion criteria and follow-up period along the three RCTs; two RCTs were monitored for up to 12 months [11, 21], and one RCT up to 36 months [13]. Additionally, two RCTs were single-center [11, 13], and one RCT was multi-center [21] with a small number of patients, which could raise concerns about selection bias. Further investigation and research are warranted to address these gaps and investigate the efficacy of radiofrequency versus cryoablation in PeAF with large left atrium size as Baimbetov et al. [13] excluded patients with left atrium size > 5.0 cm with no clear data in the other two RCTs.

### Implications for future research

The follow-up period was not constant between the studies, introducing an inconsistency in the duration of monitoring. Additionally, the differences in monitoring devices among the RCTs may introduce measurement bias. Furthermore, future research should explore the impact of provider experience and technological advancements on outcomes to gain a deep understanding of these contributing factors.

## Conclusions

In conclusion, RFA and CBA are comparable in efficacy and safety. However, CBA was associated with a shorter total procedure time.

### Supplementary Information

**Additional file 1**. Table S1: Search strategy.

## Data Availability

Not applicable.

## Abbreviations

AF: Atrial fibrillation
PV: Pulmonary vein
RFA: Radiofrequency ablation
CBA: Cryoballoon ablation
PeAF: Persistent atrial fibrillation
RCT: Randomized controlled trial
BMI: Body mass index
LVEF: Left ventricular ejection fraction
RR: Risk ratio
MD: Mean difference
CI: Confidence interval
