# Supplementary material for: Efficacy and safety of radiofrequency ablation versus cryoballoon ablation for persistent atrial fibrillation: a systematic review and meta-analysis of randomized controlled trials
Source: Egypt Heart J. 2024 Jul 8;76:89. doi: 10.1186/s43044-024-00518-x (PMC11231113; doi:10.1186/s43044-024-00518-x)
Supplement: Supplementary file 1 — Additional file 1. Table S1: Search strategy. [file 43044_2024_518_MOESM1_ESM.docx]

**Supplementary material:**

**Title.**

**Efficacy and Safety of Radiofrequency Ablation versus Cryoballoon Ablation for Persistent Atrial Fibrillation: A Systematic Review and Meta-analysis of Randomized Controlled Trials.**

**Contents:**

**Figure.**Figure S1: Forest plot of repeated ablation outcome.

**Tables.**Table S1: Search strategy.

Table S2: Summary characteristics (radiofrequency and cryoballoon ablation details).

Table S3: Baseline characteristics (medication history and patients comorbidities).

Table S4: Description of risk of bias (ROB) assessment.

Table S5: Sensitivity analysis.


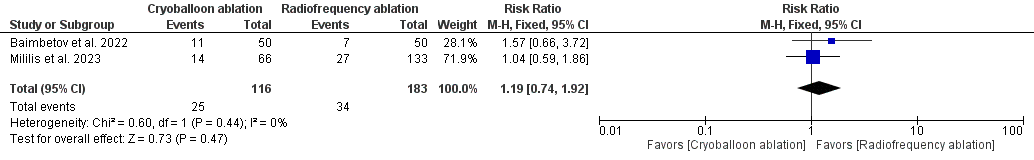


**Figure S1: Forest plot of repeated ablation outcome.**

| Database | Search Terms | Search Field | Search Results |
| --- | --- | --- | --- |
| Pubmed | ("Radiofrequency Ablation" OR "Radio‑Frequency Ablation" OR Radiofrequency OR Radio‑Frequency) AND ("Cryoballoon ablation" OR Cryoballoon) AND ("persistent atrial fibrillation*" OR "persistent afib" OR "persistent AF") | All Field | 153 |
| Cochrane | ("Radiofrequency Ablation" OR "Radio‑Frequency Ablation" OR Radiofrequency OR Radio‑Frequency) AND ("Cryoballoon ablation" OR Cryoballoon) AND ("persistent atrial fibrillation*" OR "persistent afib" OR "persistent AF") | All Field | 42 |
| WOS | ("Radiofrequency Ablation" OR "Radio‑Frequency Ablation" OR Radiofrequency OR Radio‑Frequency) AND ("Cryoballoon ablation" OR Cryoballoon) AND ("persistent atrial fibrillation*" OR "persistent afib" OR "persistent AF") | All Field | 293 |
| SCOPUS | TITLE-ABS-KEY ( ( "Radiofrequency Ablation" OR "Radio‑Frequency Ablation" OR radiofrequency OR radio‑frequency ) AND ( "Cryoballoon ablation" OR cryoballoon ) AND ( "persistent atrial fibrillation*" OR "persistent afib" OR "persistent AF" ) ) | Title, Abstract, Keywords | 205 |
| EMBASE | #4. #1 AND #2 AND #3 376  #3. 'persistent atrial fibrillation':ti,ab,kw OR 12,873  'persistent afib':ti,ab,kw OR 'persistent  af':ti,ab,kw  #2. 'cryoballoon ablation':ti,ab,kw OR 3,700  cryoballoon:ti,ab,kw  #1. 'radiofrequency ablation':ti,ab,kw OR 78,526  'radio‑frequency ablation':ti,ab,kw OR  radiofrequency:ti,ab,kw OR  radio‑frequency:ti,ab,kw | All Field | 376 |

**Table S1: Search Strategy.**

| **Study ID** | **Radiofrequency ablation** | **Cryoballoon ablation** |  |
| --- | --- | --- | --- |
|  |  |  |  |
| **Baimbetov et al. 2022** | Radiofrequency was delivered using an irrigated ThermoCool SmartTouch catheter,after a transseptal puncture with the same catheter, a 3-dimensional electroanatomic map of the LA with PVs was constructed,the RFA value was 30 W,The contact force is from 5 to 40 g, with a radio frequency application duration of 20 to 40 seconds. | Electrical isolation of the four main PVs: The transseptal puncture was performed under the control of fluoroscopy or intracardiac echocardiography, a 28-mm balloon catheter Arctic Front for cryoablation was conducted on a conductor to the PV, Cryoablation was usually performed for 240 seconds. |  |
| **Mililis et al. 2023** | A 20‐pole mapping catheter was used for the three‐dimensional reconstruction of the LA geometry with CARTO 3® navigation system,ablation was delivered via open‐tip irrigated RF catheter with tip‐integrated contact force (CF) sensor,a targeted ablation index of 400 and 500 was used on posterior and anterior wall respectively | A 28‐mm diameter cryoballoon with a circular mapping catheter was inserted in the LA through a steerable 12‐Fr sheath after a single transseptal puncture ,venography was used to confirm the complete occlusion of the PV by the balloon,an application of 240 s was performed in each PV. |  |
| **Shi et al. 2022 (NO-PERSAF)** | After the transseptal puncture, a long sheath was placed in the LA. A circular mapping catheter was inserted in the PVs for monitoring the pulmonary potentials. All patients were treated with a contact-force sensing irrigated ablation catheter with support of a deflectable long sheath,an encircling ablation strategy was performed in all PVs with targeting force-time-integral of 400 gs for each lesion. | After the transseptal puncture, a steerable 12-Fr sheath was placed in the LA,all patients were treated with a 28-mm diameter cryoballoon,a circular mapping catheter was inserted through the lumen of the cryoballoon and was advanced more distally to stabilize the cryoballoon at the PV ostium.The ablation regimen consisted of two freezing applications of 240 s in each PV, no matter the PV was isolated or not after the first CBA. |  |

**Table S2: Summary characteristics (radiofrequency and cryoballoon ablation details).**

LA: left atrium; PV:pulmonary vein; RFA: radiofrequency ablation.

| **Study ID** | **Medications N. (%)** | | | | **Comorbidities N.(%)** | | | | | | | | | |
| --- | --- | --- | --- | --- | --- | --- | --- | --- | --- | --- | --- | --- | --- | --- |
|  | **Beta-blocker** | | **Amiodarone** | | **Hypertension** | | **Diabetes** | | **IHD or CAD** | | **Obstructive sleep apnea** | | **Dyslipidemia** | |
|  | **Radiofrequency ablation** | **Cryoballoon ablation** | **Radiofrequency ablation** | **Cryoballoon ablation** | **Radiofrequency ablation** | **Cryoballoon ablation** | **Radiofrequency ablation** | **Cryoballoon ablation** | **Radiofrequency ablation** | **Cryoballoon ablation** | **Radiofrequency ablation** | **Cryoballoon ablation** | **Radiofrequency ablation** | **Cryoballoon ablation** |
| **Baimbetov et al. 2022** | NA | NA | NA | NA | NA | NA | NA | NA | NA | NA | NA | NA | NA | NA |
| **Mililis et al. 2023** | NA | NA | 20 (15) | 9 (13.6) | NA | NA | NA | NA | 53 (39.9) | 26 (39.4) | NA | NA | 20 (15) | 9 (13.6) |
| **Shi et al. 2022 (NO-PERSAF)** | 28 (58.0) | 29 (55.8) | 4 (8.2) | 1 (1.9) | 6 (12.2) | 4 (7.7) | 3 (6.1) | 5 (9.6) | NA | NA | 28 (58.0) | 29 (55.8) | 4 (8.2) | 1 (1.9) |

**Table S3: Baseline characteristics (medication history and patients comorbidities).**

IHD: ischemic heart disease; CAD: coronary artery disease; NA: not available

| **Study ID** | **Domain** | **Decision** | **Description** |
| --- | --- | --- | --- |
|  |  |  |  |
| **Baimbetov et al. 2022** | Randomization process | Some concerns | The study does not describe how data were randomized, and there were no apparent differences between the two groups. |
|  | Deviations from intended interventions | Some concerns | This stydy is open-label study. There was no deviation from the intended interventions because of the trial context. |
|  | Missing outcome data | Low risk | Outcome data of nearly all randomized patients were available. |
|  | Measurement of the outcome | Low risk | Appropriate tools were used to measure the outcome without difference between the two group arms. |
|  | Selection of the reported result | Some concerns | There are some concerns regarding whether all outcomes, measurement tools, and analysis plans were pre-specified in this study. |
|  | **OVERALL** | **SOME CONCERNS** | |
| **Mililis et al. 2023** | Randomization process | Some concerns | The study does not describe how data were randomized, and there were no apparent differences between the two groups. |
|  | Deviations from intended interventions | Some concerns | This stydy is open-label study. There was no deviation from the intended interventions because of the trial context. |
|  | Missing outcome data | Low risk | Outcome data of nearly all randomized patients were available. |
|  | Measurement of the outcome | Low risk | Appropriate tools were used to measure the outcome without difference between the two group arms. |
|  | Selection of the reported result | Some concerns | There are some concerns regarding whether all outcomes, measurement tools, and analysis plans were pre-specified in this study. |
|  | **OVERALL** | **SOME CONCERNS** | |
| **Shi et al. 2022 (NO-PERSAF)** | Randomization process | Some concerns | study did not mention how data were randomized and study was open lablel, and there were no apparent differences between the two groups. |
|  | Deviations from intended interventions | Low risk | This study was an open-label study. There was no deviation from the intended interventions because of the trial context. Additionally, the analysis was done by the intention to treat analysis. |
|  | Missing outcome data | Low risk | Outcome data were available for nearly all participants. |
|  | Measurement of the outcome | Low risk | Appropriate tools were used to measure the outcome without difference between the two group arms. |
|  | Selection of the reported result | Low risk | data that produced this result analysed in accordance with a pre-specified analysis plan. |
|  | **OVERALL** | **SOME CONCERNS** | |

**Table S4: Description of risk of bias (ROB) assessment.**

| Outcome | No. of  Participants (/) | No. of  trials | Quantitative data synthesis | | | | Heterogeneity analysis | | |
| --- | --- | --- | --- | --- | --- | --- | --- | --- | --- |
|  |  |  | MD | 95% CI | Z value | p-value | df | p-value | I2 (%) |
| **Total procedure time (min).** | | | | | | | | | |
| Baimbetov et al. 2022 | 182/118 | 2 | 49.72 | [25.60, 73.85] | 4.04 | 0.0001 | 1 | 0.003 | 89% |
| **Mililis et al. 2023** | 99/102 | 2 | 36.92 | [30.45, 43.40] | 11.18 | 0.00001 | 1 | 0.98 | 0% |
| Shi et al. 2022 (NO-PERSAF) | 183/116 | 2 | 49.11 | [25.11, 73.11] | 4.01 | 0.0001 | 1 | 0.0001 | 95% |
| **Fluoroscopy time (min).** | | | | | | | | | |
| **Baimbetov et al. 2022** | 182/118 | 2 | 0.47 | [-1.87, 2.80] | 0.39 | 0.69 | 1 | 0.35 | 0% |
| Mililis et al. 2023 | 99/102 | 2 | -5.17 | [-18.90, 8.57] | 0.74 | 0.46 | 1 | 0.00001 | 97% |
| Shi et al. 2022 (NO-PERSAF) | 183/116 | 2 | -6.27 | [-17.75, 5.21] | 1.07 | 0.28 | 1 | 0.00001 | 97% |

**Table S5: Sensitivity analysis**

MD: mean difference; CI: confidence interval; df: degrees of freedom.
